# Understanding Multiple Intelligences Among Older Adults: Development and Validation of the Multiple Intelligence Scale for Older People

**DOI:** 10.1002/brb3.71762

**Published:** 2026-09-23

**Authors:** Tzu‐Yu Lin, Ikeuchi Tomoko, I‐Hua Chen, Servet Üztemur, Mark D. Griffiths, Chung‑Ying Lin

**Affiliations:** ^1^ Master Program of Long‐Term Care in Aging, College of Nursing Kaohsiung Medical University Kaohsiung Taiwan; ^2^ Center for Long‐Term Care Research Kaohsiung Medical University Kaohsiung Taiwan; ^3^ Department of Medical Research Kaohsiung Medical University Hospital Kaohsiung Taiwan; ^4^ Department of Human Science Takachiho University Tokyo Japan; ^5^ Chinese Academy of Education Big Data, Faculty of Education Qufu Normal University Qufu China; ^6^ Department of Turkish and Social Sciences Education, Faculty of Education Anadolu University Eskişehir Türkiye; ^7^ Nottingham Institute of Psychology Nottingham Trent University Nottingham UK; ^8^ Institute of Allied Health Sciences, College of Medicine National Cheng Kung University Tainan Taiwan; ^9^ Biostatistics Consulting Center, National Cheng Kung University Hospital, College of Medicine National Cheng Kung University Tainan Taiwan; ^10^ Department of Occupational Therapy, College of Medicine National Cheng Kung University Tainan Taiwan; ^11^ Department of Public Health, College of Medicine National Cheng Kung University Tainan Taiwan

**Keywords:** cognitive health, geriatric assessment, older adults, psychometrics

## Abstract

**Background:**

Previous studies have supported a multidimensional view of intelligence, particularly the concept of multiple intelligence (MI). However, the application and comprehensive understanding of MI in the context of aging have been explored infrequently. The present study aimed to develop and validate the Multiple Intelligence Scale for Older People (MISFOP) for community‐dwelling older adults, incorporating spiritual intelligence and ensuring cultural sensitivity through translation, adaptation, and psychometric validation.

**Methods:**

The research comprised two studies to develop and validate the Chinese MISFOP. Study 1 involved translation and adaptation through expert review and pilot testing. Study 2 evaluated the 36‐item instrument with 428 older adults using exploratory structural equation modeling (ESEM) and correlations with the Montreal Cognitive Assessment (MoCA).

**Results:**

ESEM resulted in a 29‐item scale comprising an eight‐factor structure, merging intrapersonal and spiritual intelligences due to theoretical insights and statistical considerations. The revised model showed excellent fit using the comparative fit index (CFI = 0.991), non‐normed fit index (NNFI = 0.982), root mean square error of approximation (RMSEA = 0.020), and standardized root mean square residual (SRMR = 0.017), with high internal consistency for the total scale (Cronbach's *α* = 0.91). Significant positive associations were found between MoCA scores and linguistic (*β* = 0.36, *p* < 0.001), logical–mathematical (*β* = 0.26, *p* = 0.007), and the combined intrapersonal/spiritual intelligence (*β* = 0.23, *p* = 0.001).

**Conclusion:**

The findings suggest associations between specific MI domains and cognitive health, indicating potential applications in geriatric care. Moreover, further research with larger, longitudinal samples across different cultural contexts is necessary.

## Graphical abstract

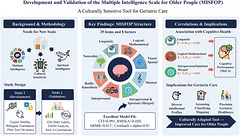

## 1 Introduction

Multiple intelligence (MI) comprises the biopsychological abilities that process information activated within a cultural context to solve problems or create valuable products (Gardner 1999; Shearer 2012). MI recognizes the diverse ways individuals exhibit intellectual strengths across various domains, fostering personal growth and a deeper appreciation of self and others throughout the lifespan (Gardner 2011). Foundational psychometric theories include Spearman's (1904) early 20th‐century two‐factor model, which categorized intelligence into a general factor (“*g*,” indicating general intelligence) predictive of academic achievement and specific factors. Subsequent research further refined the *g* factor, distinguishing between general fluid (G*f*) and crystallized (G*c*) intelligence based on empirical testing outcomes (Horn and Cattell 1966). The *g* factor represents general mental capacity relevant to educational development, often assessed through standardized tests such as the fourth version of the Wechsler Adult Intelligence Scale (WAIS‐IV). However, the measurement may result in underestimated results among older adults. Other developed models of intelligence, such as Carroll's three‐stratum theory (Carroll 1993) and Gardner's MI theory (Gardner 1983, 1999, 2011), acknowledge the diverse nature of human intelligence, encompassing diverse abilities beyond traditional cognitive skills (Lin et al. 2025). Importantly, intelligence is not static; environmental influences, individual learning experiences, and health status can shape it in older age (Andrzejewski et al. 2026; Kobayashi 2025; Ma et al. 2025). In fact, several intelligences may simultaneously exist or be complementary to perform functional ability. Therefore, older adults can leverage multiple domains to enhance their functional abilities and confidence in equal measure (Gardner 1999; Leanos et al. 2023).

The MI theory was developed by Gardner (1983, 1999, 2011), and encompasses eight diverse intelligences, including linguistic, logical–mathematical, visual–spatial, bodily–kinesthetic, musical, interpersonal, intrapersonal, and naturalist intelligence. Each intelligence is independent, flexible, and evidence‐based (Armstrong 2009). Because intelligence represents an individual's mental ability to think, learn, and solve problems in their daily lives, the development of MI depends on culturally‐valued activities and participation from early childhood to old age. Each of MI's growth trajectories differs between the lifespans and individuals. Previous studies noted that academic intelligence, mainly linguistic and logical–mathematical intelligence, develops in early life and may gradually decline in later life (Castejon et al. 2010; Visser et al. 2006; Waterhouse 2023; Winter et al. 2025). However, the other intelligences develop across the lifespan and are learned from life experiences, and develop in diverse patterns (Armstrong 2009). Nevertheless, the debate on whether the structure of MI belongs to the *g* factor or is separately independent remains ongoing. There is also a ninth type of intelligence—spiritual intelligence (SI)—which is rarely included in the examined model. Most importantly, MI studies rarely include older adults (Lin et al. 2025).

Studies examining SI have increased since the end of the 1990s, inspired by Gardner's (1999) work. Gardner (2000) is unsure whether SI is a type of intelligence due to a lack of neurological evidence, and because it relies on feeling experience, and is inadequate in explaining how the brain processes this information while problem solving. However, other evidence‐based studies have proposed that SI is a separate type of intelligence from intelligence quotient (IQ) and emotional quotient (EQ) (Emmons 2000; Skrzypińska 2021). These studies argue that SI involves unique cognitive abilities that enable individuals to solve problems and live more fulfilling lives. However, other studies claim that SI may be a set of skills and abilities rather than a type of intelligence (Mahmood et al. 2018; Pinto et al. 2023; Skrzypińska 2021).

SI is a multifaceted concept, and currently without an agreed upon definition. Most studies converge on the idea that it involves four domains, comprising critical existential thinking, personal meaning production, transcendental awareness, and conscious state expansion (Pinto et al. 2024; Zohar and Marshall 2000). SI develops by nurture, is learned by practices and experiences, increases with age (Pinto et al. 2024), and has a significant relationship with positive mental outcomes, such as lower depression, higher well‐being, greater resilience, and higher emotional intelligence among students, healthcare professionals, and older adults (Pinto et al. 2023; Pinto et al. 2024; Roy et al. 2021; Skrzypińska 2021). Therefore, integrating the SI to assess MI among older adults is necessary and crucial.

Current empirical studies and assessments of MI have largely focused on preadolescent, adolescent, and adult populations. Key assessments such as the Teele Inventory of MI (TIMI) (Teele 1992, 1996), the MI Developmental Assessment Scale (MIDAS) (Shearer 1999), Armstrong's 80‐item checklist of MI (Armstrong 2009; Lii and Wong 2010), and the MI Profiling Questionnaire IX (MIPQ IX) (Tirri and Nokelainen 2008, 2011; Tirri et al. 2013) have contributed valuable insights. However, their reliability and validity warrant further exploration. Consequently, the assessment of MI among older adults remains ambiguous and lacks a standardized method (Waterhouse 2023). To address this gap, Lin et al. (2025) developed the 64‐item Chinese MI Scale (MIS) specifically for community‐dwelling older adults and confirmed its construct validity using exploratory structural equation modeling (ESEM). However, the MIS presents several limitations, including numerous items for older adults, low factor loadings for some dimensions, not being specifically designed for older adults, and lack of the ninth intelligence (i.e., SI). This highlights the urgent need for further investigation to enhance the effectiveness of MI assessments among older adults and to ensure that their unique intelligence is accurately recognized and nurtured.

The present study aimed to adapt and integrate the appropriate structure of the new scale, the MIS for Older People (MISFOP), through two studies: translating and adapting the MIS (Study I) and establishing its reliability and validity (Study II).

## 2 Methods

### 2.1 Procedure and Participants

Study I: Adapting the MIPQ IX into Chinese for older adults. The first study utilized Jones et al.’s (2001) modified back‐translation model for cross‐cultural adaptation and translation, following a structured six‐step method to ensure content validity (Appendix A). The study adapted the MIPQ IX to develop the MISFOP due to its wide application in students and adults. The original 35‐item MIPQ IX was developed with 183 preadolescents, 86 adolescents, and 227 adults, and evaluated its validity and other psychometric properties (Tirri and Nokelainen 2011). The MIPQ IX was reported to have fair‐to‐good internal consistency for the adult population, with Cronbach's *α* coefficients ranging from 0.54 to 0.89 across the nine dimensions (Tirri and Nokelainen 2011).

After receiving permission from the original author of MIPQ IX to adapt the scale for older people, a total of six bilingual experts were invited to participate in the translation and adaptation process. Additionally, nine older participants (six males and three females aged 65–72 years) were invited to revise the items’ content in a pilot study. The pilot scale was provided to them, and their feedback was recorded.

The final version of the MISFOP consists of 36 items, comprising nine domains of MI with four items for each domain (Appendix B). Participants rate their agreement with the intelligent strengths or weaknesses in each MI domain. Items are rated using a five‐point Likert scale from 1 (strongly disagree) to 5 (strongly agree), and higher scores in each domain indicate higher intelligence in that domain. The MISFOP subscale score is calculated based on the sum of the four items in each domain.

Study 2: MISFOP verification using Chinese samples. To assess the reliability and validity of the MISFOP, the study utilized a cross‐sectional design and included a convenience sample of 428 community‐dwelling individuals aged 65 years and older. After analyzing the results, the final version of the MISFOP was revised accordingly and as a psychometric instrument tailored for older adults in their communities.

Participants were recruited from 20 communities and senior centers across five regions in Kaohsiung City, Southern Taiwan, from May to October 2024. The inclusion criteria for participants were (i) being older adults (aged 65 years or older on the recruitment date), (ii) having the ability to read and write in Chinese, (iii) having the ability to walk independently, and (iv) having a score of 18 or higher on the Montreal Cognitive Assessment (MoCA) (see “Measures” below). Individuals were excluded from the study if they had a disability or severe illnesses, such as cancer, chronic kidney disease, or spinal cord disease. Out of the original 429 older adults who met the inclusion criteria, 428 participants fully participated in the assessment, with one participant quitting, resulting in a response rate of 99.77%.

The recruitment process involved directly contacting social workers and leaders from community and senior centers by telephone. After receiving their agreement to assist in recruiting participants, research team members directly administered the survey and conducted physical and cognitive measurements to older adults. All participants provided written informed consent, and agreed to participate in the present study. The study was approved by the Institutional Review Board of Kaohsiung Medical University Chung‐Ho Memorial Hospital (#KMUHIRB‐F(II)‐20230014).

The target sample size was derived from the participant‐to‐item ratio conventionally applied in factor‐analytic scale development, because no prior parameter estimates were available for the MISFOP. A minimum of 10 participants per item (Nunnally and Bernstein 1994) results in a target of 360 for the 36‐item pool, and the attained sample of 428 corresponds to a ratio of 11.9:1. Its adequacy was subsequently verified by a Monte Carlo simulation (Muthén and Muthén 2002), in which samples of 150–600 were generated from the estimated measurement and structural model and evaluated against the conventional criteria of relative parameter bias below 10%, an empirical Type I error rate near the nominal 0.05, and power of at least 0.80. All three criteria were met from 250 participants upward, so the attained sample comfortably exceeded the required minimum. The full simulation is reported in Table S4.

### 2.2 Measures

In addition to collecting demographic information (i.e., sex, age, marital status, educational background, household composition, and career history, as well as alcohol and tobacco use), the survey comprised two validated psychometric assessment instruments.

#### 2.2.1 The 36‐Item Version of the MISFOP

The 36‐item MISFOP developed in the first study was used to assess MI. The reliability and validity of the scale is reported in the “Results” section (below).

#### 2.2.2 The MoCA

The MoCA (Nasreddine et al. 2005; Chinese version: Tsai et al. 2012) was used to assess mild cognitive impairment (MCI) among older adults in the community, and was administered face‐to‐face by trained research team members rather than self‐completed. The MoCA has high sensitivity and specificity for detecting MCI, and was used in the study to assess short‐term memory, visuospatial abilities, executive functions, attention, concentration, and working memory, language, and orientation. The total score is 30, and participants with a MoCA score below 26 are defined as having MCI. In the present study, the *α* coefficient was 0.74.

### 2.3 Participants

As aforementioned, the sample comprised 428 older adults, ranging in age from 65 to 89 years (mean = 72.71, SD = 4.98). Most participants were female (*n* = 343; 80.1%) rather than male (*n* = 85; 19.9%). Regarding marital status, the majority were married (*n* = 253; 59.1%), followed by divorced or widowed individuals (*n* = 159; 37.1%). Only a small number reported being single (*n* = 16; 3.7%). Educational attainment varied among participants: 152 had no formal education or had completed primary or junior high school education (35.5%), 146 had completed senior high school (34.1%), 118 had a bachelor's degree (27.6%), and 12 had a master's or doctoral degree (2.8%).

Regarding household composition, 87 participants lived alone (20.3%), 137 lived with a spouse (32.0%), 197 lived with their children or grandchildren (46.0%), and seven reported other living arrangements (1.6%). In terms of prior career history, participants most commonly came from military, civil service, or education (*n* = 114; 26.6%), followed by the service industry (*n* = 88; 20.6%), industrial or manual work (*n* = 72; 16.8%), homemaking (*n* = 71; 16.6%), commerce (*n* = 52; 12.1%), other occupations (*n* = 27; 6.3%), and agriculture, fishing, or animal husbandry (*n* = 4; 0.9%). Most participants reported no current tobacco use (*n* = 394; 92.1%) or alcohol consumption (*n* = 352; 82.2%). Percentages are rounded to one decimal place and may not total 100% exactly.

### 2.4 Statistical Analysis

All analyses were conducted in jamovi (version 2.7.38), which implements structural equation models (SEMs) through the R package lavaan. Models were estimated by maximum likelihood with robust standard errors and a scaled test statistic (MLR), which accommodates departures from multivariate normality in the five‐point Likert responses. The fit statistics reported below are therefore the robust values. ESEMs were rotated obliquely using geomin rotation, which was chosen because the study was exploratory in intent and the pattern of cross‐loadings was not specified in advance; geomin also permits correlated factors, as expected among intelligence domains (Asparouhov and Muthén 2009). The dataset was complete, with all 428 participants responding to every MISFOP and MoCA item, so no missing‐data procedure was required.

The analyses proceeded in five steps. Because the MISFOP is a self‐report instrument, the potential for common method bias was first assessed using Harman's single‐factor test, following Podsakoff et al. (2003). The variance explained by the first unrotated factor was evaluated against the 50% threshold. However, Harman's test is a diagnostic of limited sensitivity. It detects only pronounced method variance and not partial method effects out of the estimated parameters (Podsakoff et al. 2003). Therefore, the outcome of this test is treated as a preliminary check rather than as evidence that method bias is absent. Stronger procedural and statistical remedies were not applied because the present study did not test relationships among multiple self‐reported constructs, where common method variance is most consequential. The MISFOP items were examined for their internal structure rather than as predictors of another self‐reported outcome, and the sole criterion variable, the MoCA, was administered by trained research team members as a performance‐based cognitive assessment rather than self‐reported. Therefore, the MI‐to‐MoCA associations were unlikely to be inflated by common method bias arising from a shared response format.

Second, descriptive statistics, including means and standard deviations (SDs), were calculated for each intelligence domain and the MoCA scores. Additionally, Pearson correlation coefficients were calculated to examine the relationships between mean scores across intelligence domains and MoCA performance.

Third, several factor structures were systematically compared to examine the underlying structure of the 36‐item MISFOP. Analysis began with the most parsimonious unidimensional model, followed by an oblique nine‐factor model representing distinct intelligence domains. A higher‐order structure was then tested to determine whether a global intelligence construct could account for correlations among first‐order factors. However, because higher‐order models impose potentially unrealistic constraints (i.e., indirect relationships between items and the higher‐order factor with constant loadings of first‐order factors), a bifactor model was also evaluated (Swami et al. 2023). The bifactor approach offers a more flexible alternative by partitioning item covariance into (i) a global component (G‐factor) explaining variance shared across all items and (ii) specific factors (S‐factors) accounting for residual covariance within item subsets not explained by the global component (Rodriguez et al. 2016a, 2016b).

Building on these traditional approaches, ESEM was incorporated, which has recently emerged as particularly suitable for multidimensional scales such as the one examined in the present study (Marsh and Alamer 2024; Swami et al. 2023; van Zyl and ten Klooster 2022; Zhou et al. 2024). Unlike conventional CFA, ESEM addresses key limitations by allowing small cross‐loadings that better reflect the complexity of psychological measures, resulting in improved model fit and more realistic inter‐factor correlations without requiring post hoc modifications (Alamer 2022).

To determine overall model adequacy, general model fit evaluation criteria followed established guidelines, with acceptable fit indicated by comparative fit index (CFI) and non‐normed fit index (NNFI) values ≥ 0.90, root mean square error of approximation (RMSEA) values ≤ 0.08, and standardized root mean square residual (SRMR) values ≤ 0.08 (Kline 2023).

For the comparative analysis, a structured approach to model evaluation was followed. According to Alamer (2022), ESEM is preferable to the oblique nine‐factor CFA if improvements in fit indices (ΔRMSEA/ΔSRMR ≥ 0.015 and ΔCFI/ΔNNFI ≥ 0.01) are observed, especially if ESEM also resolves problems of excessively high inter‐factor correlations while maintaining strong primary factor loadings (should be > 0.40), without interpretability issues from cross‐loadings. Where the ESEM solution indicated that items should be removed, two criteria were applied. The primary criterion was a standardized primary loading below 0.40. In addition, items were evaluated for their effect on factor definition. An item was removed if retaining it produced a degenerate factor (i.e., one in which a single item accounted for nearly all of the factor variance while the remaining items of that factor fell to negligible loadings). Upon establishing adequate fit for ESEM, the bifactor ESEM was tested to represent the scale's structure comprehensively. Discriminant validity of the retained solution was evaluated through the magnitude of the latent factor correlations, with correlations below 0.85 taken to indicate that two factors are empirically distinguishable (Kline 2023). The Fornell and Larcker (1981) comparison was not applied because it is defined for independent‐clusters models, whereas ESEM estimates cross‐loadings by design.

Fourth, the reliability of the retained factor structure was examined. Internal consistency was evaluated using Cronbach's *α*, computed from the observed item responses. Composite reliability (CR) and average variance extracted (AVE) were computed from the standardized primary loadings of the retained solution. Because CR is estimated from a congeneric measurement model, it is numerically equivalent to McDonald's *ω* and is therefore reported as a single coefficient. Values of 0.70 or above were regarded as acceptable for *α* and CR, and values between 0.60 and 0.70 as marginal for subscales with few items. AVE was evaluated against the conventional 0.50 criterion (Kline 2023). For any domain retaining only two items, the Spearman–Brown coefficient is reported in place of *α*, which is not appropriate for two‐item scales. It should be noted that CR and AVE treat the complement of the squared standardized loading as item uniqueness, which holds when each item loads on a single factor. Under ESEM, where cross‐loadings are estimated by design, variance assigned to nontarget factors is charged to error. Therefore, both indices are conservative relative to their independent‐clusters counterparts.

Finally, the associations between the best‐fitting factor structure and MoCA scores were examined within aSEM framework to evaluate the external validity of the MISFOP. SET‐ESEM was employed to allow items within the MISFOP factors to flexibly cross‐load between themselves, while simultaneously restricting them from loading onto the MoCA factor. This methodological approach acknowledges the conceptual distinction between general cognitive assessment (MoCA) and specific intelligence domains, while still capturing the nuanced interrelationships between the intelligence factors. This strategy enabled the assessment of relationships between distinct intelligence domains and cognitive performance while preserving the measurement integrity (considering measurement error) of the MISFOP. Model fit was evaluated initially, and then the specific associations between the intelligence factors and MoCA scores were examined.

As a supplementary analysis, measurement invariance of the retained eight‐factor structure was examined across sex and educational level using multigroup ESEM. Configural, metric, and scalar models were compared using changes in approximate fit indices, with invariance retained when ΔCFI ≤ 0.010, ΔRMSEA ≤ 0.015, and ΔSRMR ≤ 0.030 (Chen 2007).

## 3 Results

The EFA showed that the first factor explained 29.90% of the total variance, which is substantially below the 50% threshold. This finding indicated that common method bias did not significantly influence the results of the present study. Table 1 presents descriptive statistics for the nine intelligence domains and their Pearson correlations. The means ranged from 12.78 (logical–mathematical intelligence, SD = 2.58) to 15.12 (spiritual intelligence [SI], SD = 2.48). Results indicated significant differences among these intelligence domains (*F* = 83.74, *p* < 0.001). Post hoc comparisons using Bonferroni correction showed that intrapersonal, spiritual, and naturalist intelligence demonstrated significantly higher levels than the other six intelligence domains.

**TABLE 1:** Descriptive statistics and Pearson correlations among the nine types of multiple intelligences and Montreal Cognitive Assessment (MoCA) score.

	*M* (SD)	1	2	3	4	5	6	7	8	9	10
1. Linguistic intelligence	13.09 (3.11)	—									
2. Logical–mathematical intelligence	12.78 (2.58)	0.45^***^	—								
3. Visual–spatial intelligence	14.38 (2.17)	0.45^***^	0.49^***^	—							
4. Bodily–kinesthetic intelligence	14.57 (2.27)	0.38^***^	0.34^***^	0.44^***^	—						
5. Musical intelligence	13.56 (3.13)	0.29^***^	0.28^***^	0.41^***^	0.50^***^	—					
6. Interpersonal intelligence	14.47 (2.54)	0.26^***^	0.29^***^	0.35^***^	0.45^***^	0.36^***^	—				
7. Intrapersonal intelligence	15.01 (2.47)	0.52^***^	0.34^***^	0.47^***^	0.45^***^	0.30^***^	0.44^***^	—			
8. Spiritual intelligence	15.12 (2.48)	0.56^***^	0.32^***^	0.51^***^	0.49^***^	0.34^***^	0.38^***^	0.79^***^	—		
9. Naturalist intelligence	15.08 (2.14)	0.42^***^	0.25^***^	0.42^***^	0.46^***^	0.35^***^	0.40^***^	0.56^***^	0.57^***^	—	
10. MoCA	26.57 (3.10)	0.35^***^	0.26^***^	0.11^*^	0.10^*^	0.09	0.08	0.25^***^	0.21^***^	0.10^*^	—

^*^ *p* < 0.05; ^**^ *p* < 0.01; ^***^ *p* < 0.001.

The correlation analysis showed significant positive associations between all the intelligence domains, with coefficients ranging from 0.25 to 0.79. Most intelligence domains showed significant positive correlations with MoCA scores, with the exception of musical and interpersonal intelligence, which did not reach statistical significance. Linguistic intelligence exhibited the strongest association with MoCA (*r* = 0.35, *p* < 0.001), followed by logical–mathematical (*r* = 0.26, *p* < 0.001), intrapersonal (*r* = 0.25, *p* < 0.001), and spiritual intelligences (*r* = 0.21, *p* < 0.001). While significantly correlated with MoCA scores, visual–spatial, naturalist, and bodily–kinesthetic intelligences showed weaker associations (*r* = 0.11, *p* = 0.02; *r* = 0.10, *p* = 0.04; and *r* = 0.10, *p* = 0.04, respectively).

Table 2 presents various factor structures and their respective model fit indices. Results indicated that both the unidimensional factor structure and the higher‐order factor structure failed to achieve acceptable model fit. In contrast, the oblique nine‐factor structure demonstrated acceptable fit. Notably, the bifactor model also fell short of acceptable standards, with NNFI values below 0.90.

**TABLE 2:** Model fit across different factor structures.

	*χ* ^2^ (df)	CFI	NNFI	SRMR	RMSEA (90% confidence interval)
One factor	2599.69 (594)	0.604	0.580	0.089	0.089 (0.086–0.092)
Oblique nine factor	976.57 (558)	0.917	0.907	0.055	0.042 (0.038–0.046)
Higher‐order factor	1174.14 (585)	0.884	0.875	0.069	0.049 (0.045–0.052)
Bifactor	1063.55 (558)	0.900	0.887	0.060	0.046 (0.042–0.050)
**ESEM‐nine factor**	**521.05 (342)**	**0.965**	**0.935**	**0.022**	**0.035 (0.029–0.041)**
**ESEM‐eight factor**	**236.67 (202)**	**0.991**	**0.982**	**0.017**	**0.020 (0.005–0.029)**
Bifactor ESEM	457.01 (315)	0.972	0.944	0.019	0.032 (0.026–0.039)

*Note*: The ESEM model shown in bold indicates the best representative model in the present study.

Abbreviations: CFI = comparative fit index, ESEM = exploratory structural equation modeling, NNFI = non‐normed fit index, RMSEA = root mean square error of approximation, SRMR = standardized root mean square residual.

Most significantly, the ESEM approach yielded superior model fit, with improvements over the oblique nine‐factor model in CFI (*Δ* = 0.048), NNFI (*Δ* = 0.028), RMSEA (*Δ* = 0.007), and SRMR (*Δ* = 0.033). Although the RMSEA improvement did not reach the 0.015 threshold specified by Alamer (2022), the criteria for CFI, NNFI, and SRMR were all satisfied, and ESEM additionally resolved the excessively high inter‐factor correlations observed in the oblique nine‐factor solution. Because ESEM demonstrated acceptable fit, the bifactor ESEM was also examined (see Table 2) and demonstrated satisfactory model fit. However, it did not provide sufficient improvement compared to the standard ESEM. Based on the parsimony principle, the bifactor ESEM was not considered a viable option for representing the scale structure.

Further analysis showed that factor correlations were substantially reduced from 0.29 to 0.96 in the oblique nine‐factor structure (see Table S1) to −0.11 to 0.62 in the ESEM solution (see Table S2). Importantly, many of the originally significant positive relationships between different intelligence domains became nonsignificant in the ESEM model.

Factor loadings were examined for each item obtained from ESEM, as shown in Table 3. Results showed that items from intrapersonal intelligence cross‐loaded onto SI with coefficients higher than their loadings on the primary factor. This pattern suggested considerable overlap between these two factors, indicating they could potentially be combined into a single dimension. Additionally, several items demonstrated primary factor loadings below 0.40 (e.g., Item 2 of logical–mathematical intelligence).

**TABLE 3:** Factor loadings derived under the exploratory structural equation modeling (ESEM) factor structure.

	LI	LMI	VSI	BKI	MUI	INPI	INRI	SI	NI
LI									
1. Writing is my natural way of expressing myself.	**0.81**	−0.08	0.01	−0.02	0.03	0.05	−0.09	−0.02	0
2. Knowledge in Mandarin or social studies was easier for me than mathematics, physics, and chemistry.	**0.56**	0.1	−0.01	−0.23	0.04	−0.01	−0.02	0	0.33
3. I have recently written something that I am particularly proud of or recognized for.	**0.76**	0.01	0.31	0.03	−0.08	0.01	0.01	0.02	−0.20
4. Metaphors and vivid verbal expressions help me learn effectively.	**0.57**	−0.01	0.14	−0.19	−0.01	−0.03	0.04	0.01	0.21

LMI									
5. I am good at math, physics, and chemistry.	0.16	**0.62**	0.1	−0.01	0.06	−0.03	0.02	0.02	−0.01
6. I can solve complex problems, such as learning 3C products.	0.01	**0.32**	0.36	−0.02	0	0.02	0.02	−0.01	0.16
7. I find mental arithmetic easy.	0.13	**0.55**	0.01	−0.01	0.07	0	0.03	−0.04	0.16
8. I am good at problem solving and games that require logical thinking. For example: puzzle games, checkers, Gomoku, Chinese chess, bridge, mahjong, board games, and sudoku.	0.29	**0.44**	0.19	0.09	−0.03	0.08	−0.01	−0.11	0.01

VSI									
9. I am good at geometry and subjects that involve spatial perception. For example: identify orientation on the street or the location on the floor.	−0.17	0.26	**0.48**	0	0	−0.01	−0.1	0.01	0.02
10. I can easily sort through complex and multifaceted problems.	−0.01	0.12	**0.70**	0.04	−0.06	0.01	−0.01	−0.06	0.02
11. I can easily imagine landscapes from a bird's‐eye view.	−0.08	0.01	**0.72**	−0.13	0.01	0.02	−0.12	0.06	−0.01
12. When I read, I form pictures or visual images in my mind.	0.21	0	**0.47**	−0.04	0.02	−0.01	−0.21	0.17	0

BKI									
13. I can easily use my hands to complete practical tasks. For example: cooking, baking, gardening, assembling, repairing, knitting, or sewing.	0	−0.07	0.41	**0.45**	0	−0.09	0.03	−0.03	0.01
14. I am good at demonstrating hands‐on operations to others.	0.01	0	0.43	**0.53**	0.06	0	−0.08	0.01	−0.05
15. I am good at physical activities or sports. For example: dancing, playing ball, running, or swimming.	0	0.08	0.03	**0.25**	0.21	0.06	0	0.07	0.21
16. I am good at expressing ideas through body movements.	0.21	0.05	−0.02	**0.20**	0.22	0.09	−0.12	0.02	0.27

MUI									
17. After hearing a tune once or twice, I can sing or hum it accurately.	0	−0.04	−0.01	−0.01	**0.73**	0.01	−0.02	−0.03	0.07
18. I can distinguish the sound of each instrument and melody when I listen to music.	0.01	−0.01	−0.01	0.01	**0.74**	−0.03	−0.02	0.13	0.02
19. I can easily keep the rhythm when tapping out a melody. For example: having a good sense of rhythm.	0.07	−0.05	0.01	0.07	**0.83**	−0.05	0.04	0.01	0.01
20. I can immediately detect when a melody goes off key.	−0.02	0.02	0.03	−0.13	**0.87**	0.02	0.12	−0.01	−0.04

INPI									
21. I can easily find someone to talk to, even among a group of strangers.	0.03	−0.05	0.02	0.02	0	**0.80**	−0.02	−0.04	0.01
22. I get along easily with different types of people.	−0.01	−0.04	−0.01	−0.02	−0.02	**0.84**	0.01	0.02	−0.01
23. I can easily connect with others.	0	0.01	0.03	−0.02	0.01	**0.83**	0.02	−0.02	−0.01
24. I help the group reach a consensus during teamwork and coordination.	−0.08	0.14	−0.01	0.01	0.03	**0.44**	0.24	0.01	0.20

INRI									
25. I am able to analyze my own motivations and actions.	0.02	0	0.20	−0.01	0.02	0.25	**0.78**	0.04	0.01
26. I often reflect on my feelings and emotions and look for their causes.	0	0.13	0	0.02	−0.02	0.13	**0.17**	0.69	−0.08
27. I often spend time reflecting on important issues in life.	−0.07	0.08	−0.02	−0.01	0.01	0.01	**−0.01**	0.93	0
28. I like to read psychological, philosophical, or religious literature to enhance self‐knowledge.	0.05	0.01	0.01	−0.12	−0.03	0.12	**−0.03**	0.47	0.33

SI									
29. I find it important to spend time thinking and reflecting amid a busy daily life.	0.06	−0.02	0.14	0.04	0	0.01	0.05	**0.53**	0.07
30. Even ordinary, everyday life is full of interesting or novel things.	0.13	−0.12	0.25	0.06	0.02	0.14	0.05	**0.31**	0.07
31. I often reflect on the meaning of life.	−0.03	−0.01	0.08	−0.01	0.01	−0.02	−0.14	**0.80**	0.10
32. I recognize the importance of sharing quiet moments with others.	0.03	−0.12	0.22	−0.02	−0.09	−0.01	0.03	**0.38**	0.26

NI									
33. I like the beautiful scenery and experiences inherent in nature.	0	−0.24	0.03	0.09	−0.01	0.10	0.12	−0.03	**0.59**
34. I can quickly become familiar with the names and related information of natural features, plants, or animals.	−0.01	−0.23	0.08	0	0.03	0.08	0	0.05	**0.47**
35. To protect the environment, I am mindful of my consumption patterns. For example: using trash bags, reusable cups, or chopsticks.	0.02	0.01	0.08	0.04	0.05	0.02	−0.13	0.12	**0.38**
36. I often pay attention to and collect information related to human development and historical sites.	−0.02	0	−0.10	0.18	−0.10	−0.02	0.18	0.02	**0.43**

*Note*: The values in bold indicate the factor loadings that belong to their primary factors.

Abbreviations: BKI = bodily–kinesthetic intelligence, INPI = interpersonal intelligence, INRI = intrapersonal intelligence, LI = linguistic intelligence, LMI = logical–mathematical intelligence, MUI = musical intelligence, NI = naturalist intelligence, SI = spiritual intelligence, VSI = visual–spatial intelligence.

**FIGURE 1:**
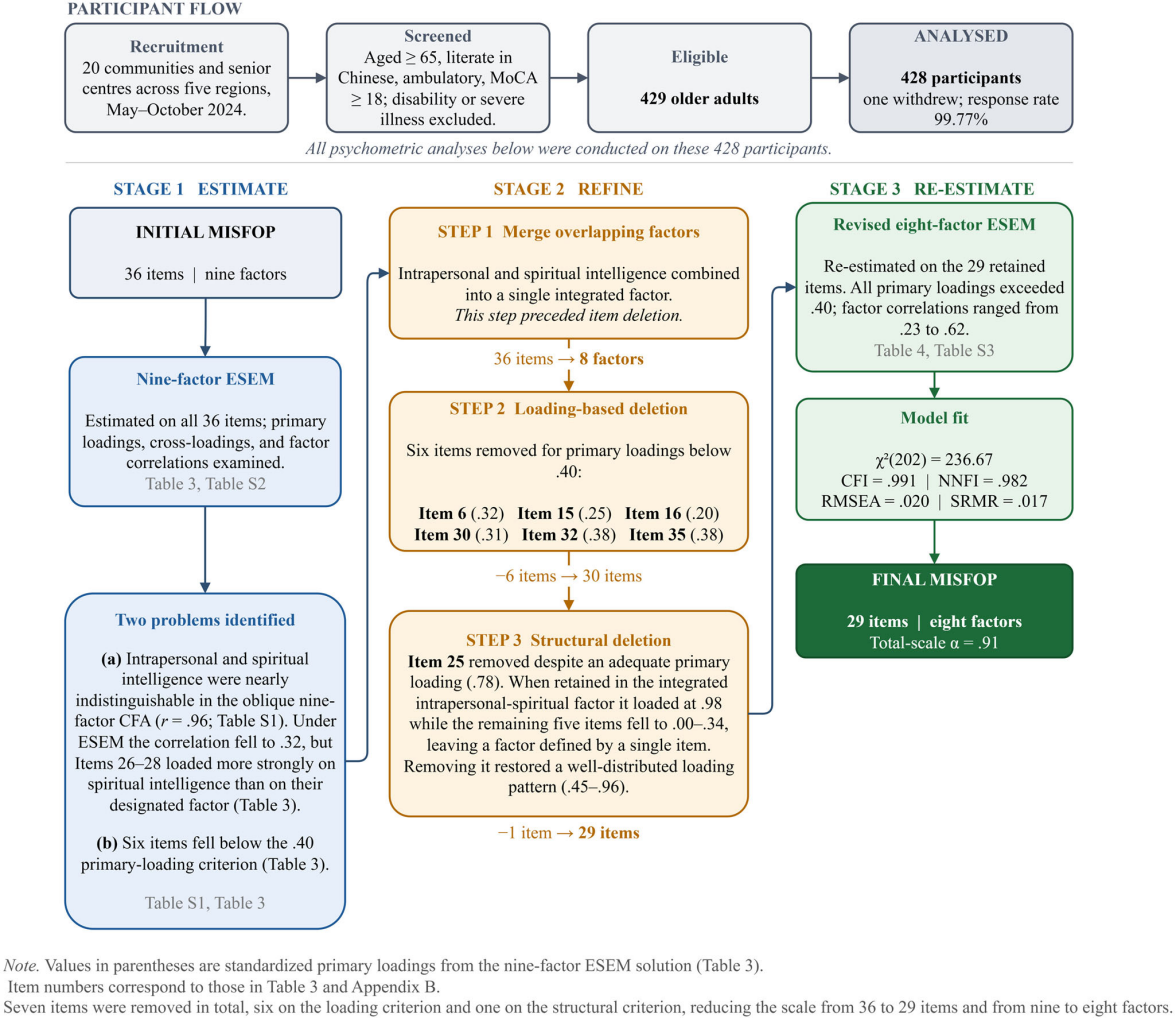
Participant flow and item‐reduction process for the MISFOP.

To address these issues, the following modifications were implemented: (i) combining intrapersonal intelligence and SI into a unified factor, and (ii) eliminating items with primary factor loadings below 0.40. The full sequence is summarized in Figure 1. First, the two factors were combined and the model was reestimated as an eight‐factor ESEM. The 0.40 criterion was then applied to this reestimated solution rather than to the original nine‐factor one, because combining the two factors changed the loadings of the items concerned. Six items met the deletion criterion (Items 6, 15, 16, 30, 32, and 35). One further item was removed on empirical grounds. When Item 25 was retained in the integrated intrapersonal–spiritual factor, it loaded at 0.98 while the remaining items of that factor dropped to between 0.00 and 0.34, so this factor was effectively defined by a single item and no longer represented the shared content of the intrapersonal and spiritual domains. Therefore, Item 25 was removed, after which the integrated factor showed a well‐distributed loading pattern (0.45–0.96; Table 4). Seven items were removed in total, reducing the scale from 36 to 29 items and from nine to eight factors.

**TABLE 4:** Revised factor loadings derived under the ESEM factor structure.

	LI	LMI	VSI	BKI	MUI	INPI	INRI + SI	NI
LI								
1. Writing is my natural way of expressing myself.	**0.86**	−0.01	−0.18	0.02	0.05	0.05	−0.01	0.02
2. Knowledge in Mandarin or social studies was easier for me than mathematics, physics, and chemistry.	**0.53**	0.20	0.03	−0.27	0.03	−0.05	−0.01	0.35
3. I have recently written something that I am particularly proud of or recognized for.	**0.78**	0.03	0.03	0.13	−0.08	0.01	0.07	−0.12
4. Metaphors and vivid verbal expressions help me learn effectively.	**0.58**	0.04	0.11	−0.17	−0.01	−0.06	0.01	0.28

LMI								
5. I am good at math, physics, and chemistry.	−0.01	**0.78**	−0.01	−0.01	0.02	−0.04	0.04	−0.08
7. I find mental arithmetic easy.	0.00	**0.61**	−0.02	−0.06	0.03	0.01	−0.03	0.09
8. I am good at problem solving and games that require logical thinking. For example: puzzle games, checkers, Gomoku, Chinese chess, bridge, mahjong, board games, and sudoku.	0.19	**0.52**	0.03	0.08	−0.05	0.10	−0.09	−0.03

VSI								
9. I am good at geometry and subjects that involve spatial perception. For example: identify orientation on the street or the location on the floor.	−0.21	0.24	**0.52**	0.06	−0.03	−0.02	−0.02	−0.06
10. I can easily sort through complex and multifaceted problems.	0.01	0.05	**0.63**	0.09	−0.06	0.02	−0.04	−0.01
11. I can easily imagine landscapes from a bird's‐eye view.	−0.07	−0.06	**0.80**	−0.04	0.00	−0.01	0.00	0.02
12. When I read, I form pictures or visual images in my mind.	0.27	−0.03	**0.44**	0.02	0.03	−0.03	0.11	−0.04

BKI								
13. I can easily use my hands to complete practical tasks. For example: cooking, baking, gardening, assembling, repairing, knitting, or sewing.	−0.01	−0.02	0.00	**0.65**	−0.02	−0.12	−0.05	0.15
14. I am good at demonstrating hands‐on operations to others.	0.01	0.03	0.05	**0.62**	0.06	0.02	0.01	−0.02

MUI								
17. After hearing a tune once or twice, I can sing or hum it accurately.	−0.01	−0.02	0.03	−0.01	**0.74**	0.03	−0.06	0.02
18. I can distinguish the sound of each instrument and melody when I listen to music.	0.00	0.01	−0.01	0.03	**0.74**	−0.02	0.10	−0.02
19. I can easily keep the rhythm when tapping out a melody. For example: having a good sense of rhythm.	0.05	−0.01	−0.09	0.13	**0.83**	−0.04	0.00	0.03
20. I can immediately detect when a melody goes off key.	−0.05	0.06	0.04	−0.05	**0.85**	0.03	−0.01	−0.03

INPI								
21. I can easily find someone to talk to, even among a group of strangers.	0.02	−0.02	0.01	0.03	0.00	**0.80**	−0.05	0.02
22. I get along easily with different types of people.	0.00	−0.02	−0.03	−0.02	−0.01	**0.85**	0.03	−0.01
23. I can easily connect with others.	−0.01	0.01	0.04	−0.03	0.00	**0.84**	−0.01	−0.02
24. I help the group reach a consensus during teamwork and coordination.	−0.13	0.16	−0.03	0.00	0.02	**0.44**	0.08	0.20

INRI + SI								
26. I often reflect on my feelings and emotions and look for their causes.	−0.02	0.09	−0.09	0.07	−0.05	0.10	**0.72**	0.00
27. I often spend time reflecting on important issues in life.	−0.05	0.00	−0.02	−0.01	0.00	−0.02	**0.96**	−0.02
28. I like to read psychological, philosophical, or religious literature to enhance self‐knowledge.	0.07	0.04	0.10	−0.15	−0.02	0.08	**0.45**	0.28
29. I find it important to spend time thinking and reflecting amid a busy daily life.	0.11	−0.05	0.05	0.05	0.02	0.01	**0.55**	0.07
31. I often reflect on the meaning of life.	0.05	−0.06	0.10	−0.02	0.04	−0.04	**0.76**	0.02

NI								
33. I like the beautiful scenery and experiences inherent in nature.	0.00	−0.13	−0.01	0.08	0.01	0.06	−0.03	**0.68**
34. I can quickly become familiar with the names and related information of natural features, plants, or animals.	0.04	−0.19	0.09	0.01	0.06	0.05	0.03	**0.50**
36. I often pay attention to and collect information related to human development and historical sites.	−0.09	0.10	−0.23	0.20	−0.13	−0.07	0.02	**0.59**

*Note*: The values in bold indicate the factor loadings that belong to their primary factors.

Abbreviations: BKI = bodily–kinesthetic intelligence, INPI = interpersonal intelligence, INRI = intrapersonal intelligence, LI = linguistic intelligence, LMI = logical–mathematical intelligence, MUI = musical intelligence, NI = naturalist intelligence, SI = spiritual intelligence, VSI = visual–spatial intelligence.

The revised structure demonstrated improved model fit: *χ* ^2^(df) = 236.67 (202), CFI = 0.991, NNFI = 0.982, RMSEA = 0.020, and SRMR = 0.017. All primary factor loadings exceeded 0.40 and most fell between 0.50 and 0.85, indicating that each domain was well defined by its retained items. Correlations between the eight factors ranged from 0.23 to 0.62 (see Table S3). The highest was well below the 0.85 criterion, supporting discriminant validity: The intelligence domains, while substantially related, remain empirically distinguishable. The decision to combine intrapersonal intelligence and SI reflects an appropriate balance between these validity considerations, recognizing when constructs are too highly correlated to be meaningfully separated. Consequently, the revised eight‐factor ESEM structure demonstrated superior fit compared to the nine‐factor ESEM solution and offered a more psychometrically sound representation of the underlying constructs.

Reliability was then examined for the revised 29‐item, eight‐factor structure, with CR and AVE computed from the standardized primary loadings reported in Table 4. Internal consistency for the total scale was high (*α* = 0.91). At the domain level, musical intelligence (*α* = 0.88, CR = 0.87), the integrated intrapersonal and spiritual intelligence domain (*α* = 0.87, CR = 0.83), interpersonal intelligence (*α* = 0.84, CR = 0.83), and linguistic intelligence (*α* = 0.84, CR = 0.79) showed good internal consistency. Logical–mathematical (*α* = 0.70, CR = 0.68) and visual–spatial intelligences (*α* = 0.69, CR = 0.70) were acceptable. Naturalist intelligence (*α* = 0.63, CR = 0.62) and bodily–kinesthetic intelligences (Spearman–Brown = 0.62, CR = 0.58) were marginal, consistent with the small number of items these domains retained after item reduction (three and two, respectively). AVE exceeded the 0.50 criterion for musical intelligence (0.63), interpersonal intelligence (0.57), and the integrated intrapersonal and spiritual intelligence domain (0.51), and closely approached it for linguistic intelligence (0.49). The remaining four domains fell below the criterion (logical–mathematical [0.42], bodily–kinesthetic [0.40], visual–spatial [0.38], naturalist [0.35]). As noted in the “Statistical Analysis” section, these values are conservative under an ESEM parameterization because cross‐loading variance is charged to item uniqueness. Linguistic intelligence illustrates this pattern, showing strong internal consistency (*α* = 0.84) alongside an AVE marginally below 0.50.

Finally, the MoCA score was incorporated alongside the revised eight‐factor structure using SET‐ESEM (which allowed items within each MISFOP factor to cross‐load onto other intelligence factors while restricting them from loading onto MoCA; see the “Statistical Analysis” section), with the MoCA score treated as an observed indicator without measurement error. The measurement model demonstrated satisfactory fit: *χ* ^2^(df) = 282.69 (223), CFI = 0.985, NNFI = 0.970, RMSEA = 0.025, and SRMR = 0.019. Significant positive associations with MoCA scores were observed for linguistic intelligence (*β* = 0.36, *t* = 4.01, *p* < 0.001), logical–mathematical intelligence (*β* = 0.26, *t* = 2.67, *p* = 0.007), and the integrated factor combining intrapersonal intelligence and SI (*β* = 0.23, *t* = 3.24, *p* = 0.001). The remaining intelligence domains (i.e., visual–spatial, bodily–kinesthetic, musical, interpersonal, and naturalist intelligences) did not demonstrate significant relationships with MoCA performance (Table 5).

**TABLE 5:** Associations between the MISFOP intelligence domains and MoCA performance from the SET‐ESEM model (*N* = 428).

			95% CI				
MISFOP factor	*B*	SE	Lower	Upper	*β*	*t*	*p*
Linguistic intelligence	1.24	0.31	0.63	1.85	0.36	4.01	< 0.001
Logical–mathematical intelligence	1.01	0.38	**0.27**	**1.75**	0.26	2.67	0.007
Visual–spatial intelligence	−0.55	0.47	−1.46	0.37	−0.18	−1.18	0.240
Bodily–kinesthetic intelligence	−0.36	0.56	−1.47	0.74	−0.12	−0.65	0.517
Musical intelligence	0.08	0.25	−0.41	0.57	0.03	0.33	0.744
Interpersonal intelligence	−0.01	0.24	−0.48	0.45	−0.01	−0.05	0.963
Integrated intrapersonal–spiritual intelligence	**0.97**	0.30	**0.38**	**1.56**	0.23	3.24	0.001
Naturalist intelligence	0.09	0.84	−1.55	1.73	0.02	0.11	0.912

*Note*: MoCA was modeled as an observed indicator without measurement error. Items were permitted to cross‐load among the MISFOP factors but were restricted from loading onto MoCA. Model fit: *χ* ^2^(223) = 282.69, CFI = 0.985, NNFI = 0.970, RMSEA = 0.025, SRMR = 0.019.

Abbreviations: CI = confidence interval, SE = standard error.

Supplementary multigroup analyses examined measurement invariance across sex and educational level (Table S5). Metric invariance was supported in both cases, as was scalar invariance across sex. Across educational level, ΔCFI (−0.011) marginally exceeded the 0.010 criterion while ΔRMSEA and ΔSRMR remained within their thresholds.

## 4 Discussion

The psychometric findings demonstrated the importance of cultural diversity in the MISFOP, because definitions and results vary across different cultural contexts (Armstrong 2009; Wilson and Mujtaba 2007). It is important to clearly distinguish each type of MI, to integrate some types of intelligence, and to enhance the understanding of MI among older adults. The present study incorporated and examined SI within the MI framework, demonstrating a positive relationship between SI and cognitive function among older adults.

The distribution of MI among older adults showed notably high scores in spiritual and naturalist intelligences, while scores for linguistic and logical–mathematical intelligences were relatively low. Previous studies examining SI have shown a positive correlation between age and SI, suggesting that older adults tend to reflect more on existential questions, seek meaning in life, and engage deeply with their spiritual beliefs and practices as they navigate life transitions and contemplate their later years (Can Oz et al. 2022; Pinto et al. 2024). In later life, the social networks of older adults do not consistently expand. Instead, some individuals may choose to withdraw from society and focus on their significant others (Carstensen 2021; Simons et al. 2023). Socioemotional selectivity theory (SST) (Carstensen 2021) provides insight into this behavior, explaining that older adults prioritize maintaining close relationships with someone they care about while distancing themselves from interactions perceived as less meaningful. The small but meaningful social networks or religiosity and spirituality (R/S) among older adults benefits their physical and mental health, such as reducing stress response, reducing depressive symptoms (Roy et al. 2021), reducing cardiovascular mortality (Shattuck and Muehlenbein 2020), increasing well‐being (Simons et al. 2023), and increasing resilience (Pinto et al. 2024). Therefore, as one of the strongest strengths of MI, the development of self‐discovery among this cohort should be considered in their lifelong learning program.

Not surprisingly, the elevated scores in naturalist intelligence indicate that older adults often maintain or strengthen their connection to the natural world. Being close to nature or immersing themselves in natural surroundings are their most common lifestyle adaptations in later life (Freeman et al. 2019), and their naturalist intelligence may gradually increase due to exposure to green or blue spaces. More specifically, empirical studies have highlighted the importance of nature‐based interventions for promoting healthy aging and indicate that spending 20–90 min for 8–12 weeks on nature‐based activities, such as gardening, hiking, or visiting parks (White et al. 2018; Coventry et al. 2021) is associated with reducing cardiovascular disease, stress, and inflammation response (Struthers et al. 2024; Wong et al. 2021). Overall, this pattern of intelligence distribution indicates that older adults’ cognitive strengths and intellectual priorities extend beyond the traditional focus on linguistic and logical–mathematical domains typically assessed in standard cognitive evaluations.

In the first study of the present study, the nine dimensions of the MISFOP were initially translated and adapted within the Chinese (Taiwanese) cultural context. The explanations for intelligence to enhance cultural relevance were also revised, primarily by straightforward descriptions and connecting the examples to their familiar life experiences. For instance, the item “I am good at problem‐solving and games that require logical thinking” includes examples of games that resonate with the Chinese culture, such as puzzle games, checkers, Gomoku, Chinese chess, bridge, mahjong, board games, and Sudoku. Additionally, the definitions of bodily–kinesthetic intelligence and naturalist intelligence were adapted to align more closely with Chinese culture (Chan 2006; Kuo et al. 2010) while staying true to the original definitions of MI theory. The original definition of bodily–kinesthetic intelligence focuses on practical skills and training by hand, whereas Chinese culture emphasizes overall kinesthetic abilities, such as mastering a sport or demonstrating hands‐on skills to others. The definition of naturalist intelligence was also revised to encompass a broader range of topics, including natural creatures, human development, and environmental protection, rather than focusing solely on environmental sensitivity (Tirri and Nokelainen 2011). Culture plays a vital role in defining and developing the concept of MI. Armstrong (2009) states, “every culture has and uses all eight intelligences” (p. 177). Therefore, it is essential to refine and adapt the explanations of each form of MI within the MISFOP framework for Taiwanese older adults. This adaptation is important for understanding and aligning with the mainstream culture that they value.

Regarding the best model of the MISFOP, the initial nine‐factor CFA model with 36 items encountered problems with high inter‐factor correlations, raising concerns about the model's discriminant validity and indicating possible overlap between the measured constructs. The subsequent application of a nine‐factor ESEM showed some improvement in these correlations. However, the increased flexibility of the model, which allows for cross‐loadings of items across factors, resulted in some previously significant factor loadings becoming nonsignificant. Ultimately, an eight‐factor ESEM with 29 items of the MISFOP was a more empirically parsimonious model that better represented the results. One significant change in the final model was integrating intrapersonal intelligence with SI, which was supported by theoretical insights (Emmons 2000; Pinto et al. 2024) and statistical considerations of factor loadings. The eight dimensions of MI in the present study provided a more comprehensive understanding among older adults. However, it is important to note that the correlation between bodily–kinesthetic intelligence and logical–mathematical intelligence was nonsignificant (Table S3). This lack of significance raises concerns about statistical power due to the limited sample size, which can inflate standard errors and make it difficult to detect actual effects.

The role of SI in MI theory is an important finding that highlights its importance in the present study. Various definitions of SI exist in the academic literature. However, many converge on the idea that it involves the ability to utilize spiritual resources and capacities for enhanced critical existential thinking, personal meaning production, transcendental awareness, and conscious state expansion (Pinto et al. 2024; Zohar and Marshall 2000). Although Gardner initially expressed concerns about including SI as a core intelligence due to the challenges of establishing scientific criteria (Gardner 2011), further studies (Bell et al. 2022; Hosseini et al. 2019; Khalsa and Newberg 2021) have reported a significant correlation between SI and cognitive function. This finding implies that SI, or at least its measurable aspects, is aligned with cognitive abilities, rather than solely a skill or trait.

A discussion on gerontological theory regarding late‐life development, known as the Gerotranscendence Theory (GTT) (Tornstam 1997, 2011), explains that psychological and spiritual development occurs in later life and supports the concept of SI among older adults. Both GTT and SI highlight the importance of self‐transcendence, meaning‐making, and a deeper understanding of cosmic or spiritual life. However, while SI encompasses a broader range of cognitive abilities related to spiritual awareness and represents an individual's capacity to solve problems, think critically, and make decisions throughout their lifespan, GTT focuses specifically on the developmental changes that occur in later life. As a result, the present study proposed and confirmed a new revised dimension of MI for older adults: the integration of intrapersonal intelligence into SI.

The present study's findings were consistent with previous studies indicating that academic intelligence—specifically linguistic and logical–mathematical intelligence—correlate with cognitive performance. Notably, the results highlighted the significant relationship between cognitive function and integrated factors that combine intrapersonal intelligence and SI. This suggests that academic intelligence does not solely determine cognitive function among older adults. Instead, a broader range of intellectual abilities plays a crucial role. Numerous studies have emphasized that academic intelligence is directly linked to core cognitive skills. While logical–mathematical intelligence may decline with age, activities such as puzzles and strategic games can help mitigate age‐related declines in fluid intelligence and strengthen the role of crystallized intelligence in sustaining cognitive performance (Harada et al. 2013; Dumas 2015; Winter et al. 2025). However, the associations observed in the present study were modest in magnitude and are likely to underestimate the true relationships. Requiring a MoCA score of 18 or above restricted the sample to the healthier end of the cognitive range (mean = 26.57, SD = 3.10), which attenuates correlations.

Surprisingly, the present study's findings showed a significant but weaker positive association between integrated factors that combine intrapersonal intelligence and SI on cognition. A systematic review showed that 82% of studies found spirituality positively affects cognitive function among middle‐aged and older adults (Hosseini et al. 2019). The present cross‐sectional study design found potential trends that, when SI was combined with intrapersonal intelligence, it may be associated with cognitive health. Researchers have found that its mechanism is influenced by enhancing resilience, self‐awareness, and meaning or purpose in life (Bell et al. 2022; Hosseini et al. 2019; Khalsa and Newberg 2021; Penman 2021). Additionally, having a sense of purpose is associated with better cognitive health, particularly for individuals aged over 50 years (Bell et al. 2022). Spirituality may help individuals cope during challenging times. For example, individuals facing serious illnesses or the end of life find that spirituality helps them maintain hope and cope more effectively (Can Oz et al. 2022; Gijsberts et al. 2011; Penman 2021). However, to advance this field and develop effective spiritually‐oriented interventions for healthy cognitive aging, it is essential to conduct longitudinal studies involving diverse populations and standardized measures of spirituality.

## 5 Limitations

The present study has several limitations that affect its findings and generalizability. First, although 86.4% of the Monte Carlo simulations fully confirmed the eight‐factor structure using the sample size in the present study (*n* = 428), a larger size may be needed to increase the confirmation rate from 86.4% to 95%. Moreover, the present sample size may be less able to detect weak relationships between the intelligence domains. Therefore, the nonsignificant correlation between bodily–kinesthetic intelligence and logical–mathematical intelligence should not be taken as evidence that the two are unrelated. This applies particularly to bodily–kinesthetic and naturalist intelligence, which retained only two and three items respectively. A larger and independent sample is needed to confirm the factor structure and to estimate the weaker associations more precisely.

Second, the sample was predominantly female (80.1%), which limits the generalizability to older males. Subgroup analysis on men was unstable due to low sample size (i.e., 85 older men, which falls far below the minimum of 250 identified in the Monte Carlo simulation) (Table S4). Additionally, although the supplementary analyses supported measurement invariance across sex, the small number of male participants suggests that such findings should be regarded as preliminary. Moreover, scalar invariance across educational level was marginally supported. Therefore, future research with larger and more gender‐balanced sample sizes is required to reevaluate measurement equivalence across sex and educational groups.

Third, reliability was established only through internal consistency at a single timepoint. Test–retest reliability and temporal stability were not assessed. Therefore, the extent to which MISFOP scores remain stable over time is unknown. Fourth, the study was cross‐sectional and correlational. Therefore, the direction of the relationship between MI and cognitive function cannot be established, Stronger intelligence profiles may support cognitive performance, and better cognition may support the activities through which these domains develop, or both may reflect a common third factor. The associations were also modest in magnitude, and their size is likely to have been attenuated by the inclusion criterion of a MoCA score of 18 or above, which restricted the sample to the healthier end of the cognitive range. Longitudinal designs and samples spanning a wider range of cognitive function are needed to clarify these relationships.

Fifth, participants were recruited by convenience sampling in a single Southern Taiwan city, using a Chinese version of the MISFOP adapted to this specific cultural context. Because cultural equivalence was central to the adaptation process, the findings may not transfer directly to other cultural or regional groups. The study also excluded individuals with disabilities or severe illnesses, which may have skewed the sample toward healthier older adults and omitted a substantial segment of the older population.

## 6 Conclusions

The present study successfully adapted and validated the MISFOP specifically for community‐dwelling older adults within a Chinese cultural context. A key finding was the integration of SI with intrapersonal intelligence into a single, robust factor, highlighting the importance of this dimension in later life. Additionally, the findings showed that older adults displayed particular strengths in spiritual and naturalist intelligence, indicating that these areas are significant aspects of intellectual engagement as individuals age. The study demonstrated that three domains of MI are significantly associated with cognitive function, as assessed by the MoCA. This supports a multidimensional view of intelligence in aging, extending beyond purely academic abilities. The validated MISFOP offers a comprehensive perspective for researchers and practitioners rethinking cognitive health in a non‐disease or non‐dysfunction approach. Moreover, the findings suggest designing an MI‐oriented and tailored intervention program according to the profiles of diverse intellectual capacities of older adults. They may show high motivation, adherence to the community program, and better performance as they age.

## Author Contributions

**Ikeuchi Tomoko**: Writing – review and editing. **Tzu‐Yu Lin**: investigation, data curation, conceptualization, writing – original draft. **I‐Hua Chen**: formal analysis, writing – original draft, data curation, methodology. **Servet Üztemur**: writing – review and editing, conceptualization, investigation. **Mark D. Griffiths**: writing – review and editing, supervision. **Chung‑Ying Lin**: writing – review and editing, methodology, supervision, conceptualization.

## Funding

This work was supported by the National Science and Technology Council under Grant 115‐2410‐H‐037‐019‐MY2, Kaohsiung Medical University research project (KMU‐TB115005), and Taishan Scholars Program Special Fund under Grant tsqn202211130.

## Ethics Statement

The research protocol received full ethical approval from the Institutional Review Board of Kaohsiung Medical University Chung‐Ho Memorial Hospital (KMUHIRB‐F(II)‐20230014). All procedures were conducted in strict accordance with established ethical guidelines for research involving human participants.

## Conflicts of Interest

The authors declare no conflicts of interest.

## Supporting information




**Supplementary Material**: brb371762‐sup‐0001‐SuppMat.docx

**Supplementary Tables**: brb371762‐sup‐0002‐TableS1‐S5.docx

## Data Availability

The data that support the findings of the present study are available from the corresponding author, I‐Hua Chen, upon reasonable request.
