# Supplementary material for: Understanding Multiple Intelligences Among Older Adults: Development and Validation of the Multiple Intelligence Scale for Older People
Source: Brain Behav. 2026 Sep 23;16(9):e71762. doi: 10.1002/brb3.71762 (PMC13601738; doi:10.1002/brb3.71762)
Supplement: Supplementary file 1 [file BRB3-16-e71762-s001.docx]

**Table Legend**

Table 1. Descriptive statistics and Pearson correlations among the nine types of multiple intelligences and Montreal Cognitive Assessment (MoCA) score.

Table 2. Model fit across different factor structures.

Table 3. Factor loadings derived under the exploratory structural equation modeling (ESEM) factor structure

Table 4. Revised factor loadings derived under the ESEM factor structure

Table 5. Standardized associations between the MISFOP intelligence domains and MoCA performance from the set exploratory structural equation modeling (SET-ESEM**)** model (*N* = 428)

**Figure Legend**

Figure 1. Participant flow and item-reduction process for the MISFOP.

**Table 1**

*Descriptive statistics and Pearson correlations among the nine types of multiple intelligences and Montreal Cognitive Assessment (MoCA) score*

|  | *M (SD)* | 1 | 2 | 3 | 4 | 5 | 6 | 7 | 8 | 9 | 10 |
| --- | --- | --- | --- | --- | --- | --- | --- | --- | --- | --- | --- |
| 1.Linguistic Intelligence | 13.09 (3.11) | — |  |  |  |  |  |  |  |  |  |
| 2.Logical-Mathematical Intelligence | 12.78 (2.58) | 0.45^***^ | — |  |  |  |  |  |  |  |  |
| 3.Visual-Spatial Intelligence | 14.38 (2.17) | 0.45^***^ | 0.49^***^ | — |  |  |  |  |  |  |  |
| 4.Bodily-Kinesthetic Intelligence | 14.57 (2.27) | 0.38^***^ | 0.34^***^ | 0.44^***^ | — |  |  |  |  |  |  |
| 5.Musical Intelligence | 13.56 (3.13) | 0.29^***^ | 0.28^***^ | 0.41^***^ | 0.50^***^ | — |  |  |  |  |  |
| 6.Interpersonal Intelligence | 14.47 (2.54) | 0.26^***^ | 0.29^***^ | 0.35^***^ | 0.45^***^ | 0.36^***^ | — |  |  |  |  |
| 7.Intrapersonal Intelligence | 15.01 (2.47) | 0.52^***^ | 0.34^***^ | 0.47^***^ | 0.45^***^ | 0.30^***^ | 0.44^***^ | — |  |  |  |
| 8.Spiritual Intelligence | 15.12 (2.48) | 0.56^***^ | 0.32^***^ | 0.51^***^ | 0.49^***^ | 0.34^***^ | 0.38^***^ | 0.79^***^ | — |  |  |
| 9.Naturalist Intelligence | 15.08 (2.14) | 0.42^***^ | 0.25^***^ | 0.42^***^ | 0.46^***^ | 0.35^***^ | 0.40^***^ | 0.56^***^ | 0.57^***^ | — |  |
| 10.MoCA | 26.57 (3.10) | 0.35^***^ | 0.26^***^ | 0.11^*^ | 0.10^*^ | 0.09 | 0.08 | 0.25^***^ | 0.21^***^ | 0.10^*^ | — |

*Note*. ^*^*p*<.05; ^**^ *p*<.01; ^***^ *p*<.001.

**Table 2**

*Model fit across different factor structures*

|  | χ^2^ (*df*) | CFI | NNFI | SRMR | RMSEA  (90% Confidence Interval) |
| --- | --- | --- | --- | --- | --- |
| One factor | 2599.69 (594) | 0.604 | 0.580 | 0.089 | 0.089 (0.086-0.092) |
| Oblique nine factor | 976.57 (558) | 0.917 | 0.907 | 0.055 | 0.042 (0.038-0.046) |
| Higher-order facor | 1174.14 (585) | 0.884 | 0.875 | 0.069 | 0.049 (0.045-0.052) |
| Bifactor | 1063.55 (558) | 0.900 | 0.887 | 0.060 | 0.046 (0.042-0.050) |
| **ESEM-Nine factor** | **521.05 (342)** | **0.965** | **0.935** | **0.022** | **0.035 (0.029-0.041)** |
| **ESEM-Eight factor** | **236.67 (202)** | **0.991** | **0.982** | **0.017** | **0.020 (0.005-0.029)** |
| Bifactor ESEM | 457.01 (315) | 0.972 | 0.944 | 0.019 | 0.032 (0.026-0.039) |

*Note*. CFI = comparative fit index; NNFI = non-normed fit index; RMSEA = root mean square error of approximation; SRMR = standardized root mean square residual. ESEM = exploratory structural equation modeling; The ESEM model shown in bold indicates the best representative model in the present study.

**Table 3**

*Factor loadings derived under the exploratory structural equation modeling (ESEM) factor structure*

|  | **LI** | **LMI** | **VSI** | **BKI** | **MUI** | **INPI** | **INRI** | **SI** | **NI** |
| --- | --- | --- | --- | --- | --- | --- | --- | --- | --- |
| LI |  |  |  |  |  |  |  |  |  |
| 1. Writing is my natural way of expressing myself. | **0.81** | -0.08 | 0.01 | -0.02 | 0.03 | 0.05 | -0.09 | -0.02 | 0 |
| 2. Knowledge in Mandarin or social studies was easier for me than mathematics, physics, and chemistry. | **0.56** | 0.1 | -0.01 | -0.23 | 0.04 | -0.01 | -0.02 | 0 | 0.33 |
| 3. I have recently written something that I am particularly proud of or recognized for. | **0.76** | 0.01 | 0.31 | 0.03 | -0.08 | 0.01 | 0.01 | 0.02 | -0.20 |
| 4. Metaphors and vivid verbal expressions help me learn effectively. | **0.57** | -0.01 | 0.14 | -0.19 | -0.01 | -0.03 | 0.04 | 0.01 | 0.21 |
| LMI |  |  |  |  |  |  |  |  |  |
| 5. I am good at math, physics, and chemistry. | 0.16 | **0.62** | 0.1 | -0.01 | 0.06 | -0.03 | 0.02 | 0.02 | -0.01 |
| 6. I can solve complex problems, such as learning 3C products. | 0.01 | **0.32** | 0.36 | -0.02 | 0 | 0.02 | 0.02 | -0.01 | 0.16 |
| 7. I find mental arithmetic easy. | 0.13 | **0.55** | 0.01 | -0.01 | 0.07 | 0 | 0.03 | -0.04 | 0.16 |
| 8. I am good at problem-solving and games that require logical thinking. For example: puzzle games, checkers, Gomoku, Chinese chess, bridge, mahjong, board games, and sudoku. | 0.29 | **0.44** | 0.19 | 0.09 | -0.03 | 0.08 | -0.01 | -0.11 | 0.01 |
| VSI |  |  |  |  |  |  |  |  |  |
| 9. I am good at geometry and subjects that involve spatial perception. For example: Identify orientation on the street or the location on the floor. | -0.17 | 0.26 | **0.48** | 0 | 0 | -0.01 | -0.1 | 0.01 | 0.02 |
| 10. I can easily sort through complex and multifaceted problems. | -0.01 | 0.12 | **0.70** | 0.04 | -0.06 | 0.01 | -0.01 | -0.06 | 0.02 |
| 11. I can easily imagine landscapes from a bird’s-eye view. | -0.08 | 0.01 | **0.72** | -0.13 | 0.01 | 0.02 | -0.12 | 0.06 | -0.01 |
| 12. When I read, I form pictures or visual images in my mind. | 0.21 | 0 | **0.47** | -0.04 | 0.02 | -0.01 | -0.21 | 0.17 | 0 |
| BKI |  |  |  |  |  |  |  |  |  |
| 13. I can easily use my hands to complete practical tasks. For example: cooking, baking, gardening, assembling, repairing, knitting, or sewing. | 0 | -0.07 | 0.41 | **0.45** | 0 | -0.09 | 0.03 | -0.03 | 0.01 |
| 14. I am good at demonstrating hands-on operations to others. | 0.01 | 0 | 0.43 | **0.53** | 0.06 | 0 | -0.08 | 0.01 | -0.05 |
| 15. I am good at physical activities or sports. For example: dancing, playing ball, running, or swimming. | 0 | 0.08 | 0.03 | **0.25** | 0.21 | 0.06 | 0 | 0.07 | 0.21 |
| 16. I am good at expressing ideas through body movements. | 0.21 | 0.05 | -0.02 | **0.20** | 0.22 | 0.09 | -0.12 | 0.02 | 0.27 |
| MUI |  |  |  |  |  |  |  |  |  |
| 17. After hearing a tune once or twice, I can sing or hum it accurately. | 0 | -0.04 | -0.01 | -0.01 | **0.73** | 0.01 | -0.02 | -0.03 | 0.07 |
| 18. I can distinguish the sound of each instrument and melody when I listen to music. | 0.01 | -0.01 | -0.01 | 0.01 | **0.74** | -0.03 | -0.02 | 0.13 | 0.02 |
| 19. I can easily keep the rhythm when tapping out a melody. For example: having a good sense of rhythm. | 0.07 | -0.05 | 0.01 | 0.07 | **0.83** | -0.05 | 0.04 | 0.01 | 0.01 |
| 20. I can immediately detect when a melody goes off key. | -0.02 | 0.02 | 0.03 | -0.13 | **0.87** | 0.02 | 0.12 | -0.01 | -0.04 |
| INPI |  |  |  |  |  |  |  |  |  |
| 21. I can easily find someone to talk to, even among a group of strangers. | 0.03 | -0.05 | 0.02 | 0.02 | 0 | **0.80** | -0.02 | -0.04 | 0.01 |
| 22. I get along easily with different types of people. | -0.01 | -0.04 | -0.01 | -0.02 | -0.02 | **0.84** | 0.01 | 0.02 | -0.01 |
| 23. I can easily connect with others. | 0 | 0.01 | 0.03 | -0.02 | 0.01 | **0.83** | 0.02 | -0.02 | -0.01 |
| 24. I help the group reach a consensus during teamwork and coordination. | -0.08 | 0.14 | -0.01 | 0.01 | 0.03 | **0.44** | 0.24 | 0.01 | 0.20 |
| INRI |  |  |  |  |  |  |  |  |  |
| 25. I am able to analyze my own motivations and actions. | 0.02 | 0 | 0.20 | -0.01 | 0.02 | 0.25 | **0.78** | 0.04 | 0.01 |
| 26. I often reflect on my feelings and emotions and look for their causes. | 0 | 0.13 | 0 | 0.02 | -0.02 | 0.13 | **0.17** | 0.69 | -0.08 |
| 27. I often spend time reflecting on important issues in life. | -0.07 | 0.08 | -0.02 | -0.01 | 0.01 | 0.01 | **-0.01** | 0.93 | 0 |
| 28. I like to read psychological, philosophical, or religious literature to enhance self-knowledge. | 0.05 | 0.01 | 0.01 | -0.12 | -0.03 | 0.12 | **-0.03** | 0.47 | 0.33 |
| SI |  |  |  |  |  |  |  |  |  |
| 29. I find it important to spend time thinking and reflecting amid a busy daily life. | 0.06 | -0.02 | 0.14 | 0.04 | 0 | 0.01 | 0.05 | **0.53** | 0.07 |
| 30. Even ordinary, everyday life is full of interesting or novel things. | 0.13 | -0.12 | 0.25 | 0.06 | 0.02 | 0.14 | 0.05 | **0.31** | 0.07 |
| 31. I often reflect on the meaning of life. | -0.03 | -0.01 | 0.08 | -0.01 | 0.01 | -0.02 | -0.14 | **0.80** | 0.10 |
| 32. I recognize the importance of sharing quiet moments with others. | 0.03 | -0.12 | 0.22 | -0.02 | -0.09 | -0.01 | 0.03 | **0.38** | 0.26 |
| NI |  |  |  |  |  |  |  |  |  |
| 33. I like the beautiful scenery and experiences inherent in nature. | 0 | -0.24 | 0.03 | 0.09 | -0.01 | 0.10 | 0.12 | -0.03 | **0.59** |
| 34. I can quickly become familiar with the names and related information of natural features, plants, or animals. | -0.01 | -0.23 | 0.08 | 0 | 0.03 | 0.08 | 0 | 0.05 | **0.47** |
| 35. To protect the environment, I am mindful of my consumption patterns. For example: using trash bags, reusable cups, or chopsticks. | 0.02 | 0.01 | 0.08 | 0.04 | 0.05 | 0.02 | -0.13 | 0.12 | **0.38** |
| 36. I often pay attention to and collect information related to human development and historical sites. | -0.02 | 0 | -0.10 | 0.18 | -0.10 | -0.02 | 0.18 | 0.02 | **0.43** |

*Note*. The values in bold indicate the factor loadings that belong to their primary factors. LI - linguistic intelligence; LMI - logical-mathematical intelligence; VSI - visual-spatial intelligence; BKI - bodily-kinesthetic intelligence; MUI - musical intelligence; INPI - interpersonal intelligence; INRI - intrapersonal intelligence; SI- spiritual intelligence; NI - naturalist intelligence

**Table 4**

*Revised factor loadings derived under the ESEM factor structure*

|  | **LI** | **LMI** | **VSI** | **BKI** | **MUI** | **INPI** | **INRI +SI** | **NI** |
| --- | --- | --- | --- | --- | --- | --- | --- | --- |
| LI |  |  |  |  |  |  |  |  |
| 1. Writing is my natural way of expressing myself. | **0.86** | -0.01 | -0.18 | 0.02 | 0.05 | 0.05 | -0.01 | 0.02 |
| 2. Knowledge in Mandarin or social studies was easier for me than mathematics, physics, and chemistry. | **0.53** | 0.20 | 0.03 | -0.27 | 0.03 | -0.05 | -0.01 | 0.35 |
| 3. I have recently written something that I am particularly proud of or recognized for. | **0.78** | 0.03 | 0.03 | 0.13 | -0.08 | 0.01 | 0.07 | -0.12 |
| 4. Metaphors and vivid verbal expressions help me learn effectively. | **0.58** | 0.04 | 0.11 | -0.17 | -0.01 | -0.06 | 0.01 | 0.28 |
| LMI |  |  |  |  |  |  |  |  |
| 5. I am good at math, physics, and chemistry. | -0.01 | **0.78** | -0.01 | -0.01 | 0.02 | -0.04 | 0.04 | -0.08 |
| 7. I find mental arithmetic easy. | 0.00 | **0.61** | -0.02 | -0.06 | 0.03 | 0.01 | -0.03 | 0.09 |
| 8. I am good at problem-solving and games that require logical thinking. For example: puzzle games, checkers, Gomoku, Chinese chess, bridge, mahjong, board games, and sudoku. | 0.19 | **0.52** | 0.03 | 0.08 | -0.05 | 0.10 | -0.09 | -0.03 |
| VSI |  |  |  |  |  |  |  |  |
| 9. I am good at geometry and subjects that involve spatial perception. For example: Identify orientation on the street or the location on the floor. | -0.21 | 0.24 | **0.52** | 0.06 | -0.03 | -0.02 | -0.02 | -0.06 |
| 10. I can easily sort through complex and multifaceted problems. | 0.01 | 0.05 | **0.63** | 0.09 | -0.06 | 0.02 | -0.04 | -0.01 |
| 11. I can easily imagine landscapes from a bird’s-eye view. | -0.07 | -0.06 | **0.80** | -0.04 | 0.00 | -0.01 | 0.00 | 0.02 |
| 12. When I read, I form pictures or visual images in my mind. | 0.27 | -0.03 | **0.44** | 0.02 | 0.03 | -0.03 | 0.11 | -0.04 |
| BKI |  |  |  |  |  |  |  |  |
| 13. I can easily use my hands to complete practical tasks. For example: cooking, baking, gardening, assembling, repairing, knitting, or sewing. | -0.01 | -0.02 | 0.00 | **0.65** | -0.02 | -0.12 | -0.05 | 0.15 |
| 14. I am good at demonstrating hands-on operations to others. | 0.01 | 0.03 | 0.05 | **0.62** | 0.06 | 0.02 | 0.01 | -0.02 |
| MUI |  |  |  |  |  |  |  |  |
| 17. After hearing a tune once or twice, I can sing or hum it accurately. | -0.01 | -0.02 | 0.03 | -0.01 | **0.74** | 0.03 | -0.06 | 0.02 |
| 18. I can distinguish the sound of each instrument and melody when I listen to music. | 0.00 | 0.01 | -0.01 | 0.03 | **0.74** | -0.02 | 0.10 | -0.02 |
| 19. I can easily keep the rhythm when tapping out a melody. For example: having a good sense of rhythm. | 0.05 | -0.01 | -0.09 | 0.13 | **0.83** | -0.04 | 0.00 | 0.03 |
| 20. I can immediately detect when a melody goes off key. | -0.05 | 0.06 | 0.04 | -0.05 | **0.85** | 0.03 | -0.01 | -0.03 |
| INPI |  |  |  |  |  |  |  |  |
| 21. I can easily find someone to talk to, even among a group of strangers. | 0.02 | -0.02 | 0.01 | 0.03 | 0.00 | **0.80** | -0.05 | 0.02 |
| 22. I get along easily with different types of people. | 0.00 | -0.02 | -0.03 | -0.02 | -0.01 | **0.85** | 0.03 | -0.01 |
| 23. I can easily connect with others. | -0.01 | 0.01 | 0.04 | -0.03 | 0.00 | **0.84** | -0.01 | -0.02 |
| 24. I help the group reach a consensus during teamwork and coordination. | -0.13 | 0.16 | -0.03 | 0.00 | 0.02 | **0.44** | 0.08 | 0.20 |
| INRI +SI |  |  |  |  |  |  |  |  |
| 26. I often reflect on my feelings and emotions and look for their causes. | -0.02 | 0.09 | -0.09 | 0.07 | -0.05 | 0.10 | **0.72** | 0.00 |
| 27. I often spend time reflecting on important issues in life. | -0.05 | 0.00 | -0.02 | -0.01 | 0.00 | -0.02 | **0.96** | -0.02 |
| 28. I like to read psychological, philosophical, or religious literature to enhance self-knowledge. | 0.07 | 0.04 | 0.10 | -0.15 | -0.02 | 0.08 | **0.45** | 0.28 |
| 29. I find it important to spend time thinking and reflecting amid a busy daily life. | 0.11 | -0.05 | 0.05 | 0.05 | 0.02 | 0.01 | **0.55** | 0.07 |
| 31. I often reflect on the meaning of life. | 0.05 | -0.06 | 0.10 | -0.02 | 0.04 | -0.04 | **0.76** | 0.02 |
| NI |  |  |  |  |  |  |  |  |
| 33. I like the beautiful scenery and experiences inherent in nature. | 0.00 | -0.13 | -0.01 | 0.08 | 0.01 | 0.06 | -0.03 | **0.68** |
| 34. I can quickly become familiar with the names and related information of natural features, plants, or animals. | 0.04 | -0.19 | 0.09 | 0.01 | 0.06 | 0.05 | 0.03 | **0.50** |
| 36. I often pay attention to and collect information related to human development and historical sites. | -0.09 | 0.10 | -0.23 | 0.20 | -0.13 | -0.07 | 0.02 | **0.59** |

*Note*. The values in bold indicate the factor loadings that belong to their primary factors. LI - linguistic intelligence; LMI - logical-mathematical intelligence; VSI - visual-spatial intelligence; BKI - bodily-kinesthetic intelligence; MUI - musical intelligence; INPI - interpersonal intelligence; INRI - intrapersonal intelligence; SI- spiritual intelligence; NI - naturalist intelligence.

**Table 5**

Associations between the MISFOP intelligence domains and MoCA performance from the SET-ESEM model (*N* = 428).

|  |  |  | 95% CI | |  |  |  |
| --- | --- | --- | --- | --- | --- | --- | --- |
| MISFOP factor | *B* | *SE* | Lower | Upper | *β* | *t* | *p* |
| Linguistic intelligence | 1.24 | 0.31 | 0.63 | 1.85 | 0.36 | 4.01 | < .001 |
| Logical-mathematical intelligence | 1.01 | 0.38 | **0.27** | **1.75** | 0.26 | 2.67 | .007 |
| Visual-spatial intelligence | -0.55 | 0.47 | -1.46 | 0.37 | -0.18 | -1.18 | .240 |
| Bodily-kinesthetic intelligence | -0.36 | 0.56 | -1.47 | 0.74 | -0.12 | -0.65 | .517 |
| Musical intelligence | 0.08 | 0.25 | -0.41 | 0.57 | 0.03 | 0.33 | .744 |
| Interpersonal intelligence | -0.01 | 0.24 | -0.48 | 0.45 | -0.01 | -0.05 | .963 |
| Integrated intrapersonal-Spiritual intelligence | **0.97** | 0.30 | **0.38** | **1.56** | 0.23 | 3.24 | .001 |
| Naturalist intelligence | 0.09 | 0.84 | -1.55 | 1.73 | 0.02 | 0.11 | .912 |

*Note.* MoCA was modeled as an observed indicator without measurement error. Items were permitted to cross-load among the MISFOP factors but were restricted from loading onto MoCA. *B* = unstandardized coefficient; *SE* = standard error; CI = confidence interval; *β* = standardized coefficient. Model fit: χ²(223) = 282.69, CFI = .985, NNFI = .970, RMSEA = .025, SRMR = .019.

**Table S1**

*Latent factor correlations among the nine intelligence domains from the oblique CFA model*

|  | 1 | 2 | 3 | 4 | 5 | 6 | 7 | 8 | 9 |
| --- | --- | --- | --- | --- | --- | --- | --- | --- | --- |
| 1.Linguistic Intelligence | — |  |  |  |  |  |  |  |  |
| 2.Logical-Mathematical Intelligence | 0.58^***^ | — |  |  |  |  |  |  |  |
| 3.Visual-Spatial Intelligence | 0.62^***^ | 0.66^***^ | — |  |  |  |  |  |  |
| 4.Bodily-Kinesthetic Intelligence | 0.52^***^ | 0.50^***^ | 0.64^***^ | — |  |  |  |  |  |
| 5.Musical Intelligence | 0.34^***^ | 0.35^***^ | 0.53^***^ | 0.65^***^ | — |  |  |  |  |
| 6.Interpersonal Intelligence | 0.29^***^ | 0.34^***^ | 0.44^***^ | 0.56^***^ | 0.41^***^ | — |  |  |  |
| 7.Intrapersonal Intelligence | 0.62^***^ | 0.42^***^ | 0.62^***^ | 0.57^***^ | 0.33^***^ | 0.45^***^ | — |  |  |
| 8.Spiritual Intelligence | 0.67^***^ | 0.41^***^ | 0.69^***^ | 0.64^***^ | 0.40^***^ | 0.41^***^ | 0.96^***^ | — |  |
| 9.Naturalist Intelligence | 0.56^***^ | 0.32^***^ | 0.60^***^ | 0.66^***^ | 0.46^***^ | 0.52^***^ | 0.70^***^ | 0.75^***^ | — |

*Note*. ^***^*p* < .001

**Table S2**

*Latent factor correlations among the nine intelligence domains from the exploratory structural equation model*

|  | 1 | 2 | 3 | 4 | 5 | 6 | 7 | 8 | 9 |
| --- | --- | --- | --- | --- | --- | --- | --- | --- | --- |
| 1.Linguistic Intelligence | — |  |  |  |  |  |  |  |  |
| 2.Logical-Mathematical Intelligence | 0.13 | — |  |  |  |  |  |  |  |
| 3.Visual-Spatial Intelligence | 0.41^*^ | 0.19 | — |  |  |  |  |  |  |
| 4.Bodily-Kinesthetic Intelligence | 0.11 | -0.08 | 0.03 | — |  |  |  |  |  |
| 5.Musical Intelligence | 0.23 | 0.09 | 0.52^**^ | 0.18 | — |  |  |  |  |
| 6.Interpersonal Intelligence | 0.23 | 0.10 | 0.47 | 0.27 | 0.41^**^ | — |  |  |  |
| 7.Intrapersonal Intelligence | 0.13 | -0.11 | 0.21 | 0.19 | -0.03 | 0.12 | — |  |  |
| 8.Spiritual Intelligence | 0.53 | 0.01 | 0.55 | 0.11 | 0.23 | 0.29 | 0.32 | — |  |
| 9.Naturalist Intelligence | 0.47 | 0.16 | 0.62^*^ | 0.11 | 0.38 | 0.42 | 0.22 | 0.54 | — |

*Note*. ^**^*p*<.05; ^**^ *p*<.01

**Table S3**

|  | 1 | 2 | 3 | 4 | 5 | 6 | 7 | 8 |
| --- | --- | --- | --- | --- | --- | --- | --- | --- |
| 1.Linguistic Intelligence | — |  |  |  |  |  |  |  |
| 2.Logical-Mathematical Intelligence | 0.42^***^ | — |  |  |  |  |  |  |
| 3.Visual-Spatial Intelligence | 0.52^***^ | 0.51^**^ | — |  |  |  |  |  |
| 4.Bodily-Kinesthetic Intelligence | 0.30^**^ | 0.23 | 0.46^*^ | — |  |  |  |  |
| 5.Musical Intelligence | 0.31^**^ | 0.26^**^ | 0.52^***^ | 0.40^***^ | — |  |  |  |
| 6.Interpersonal Intelligence | 0.26^**^ | 0.24^*^ | 0.43^***^ | 0.45^***^ | 0.39^***^ | — |  |  |
| 7.integrated Intrapersonal and spiritual Intelligence | 0.52^***^ | 0.30^**^ | 0.54^***^ | 0.36^**^ | 0.28^***^ | 0.32^***^ | — |  |
| 8.Natural Intelligence | 0.50^***^ | 0.31^*^ | 0.59^***^ | 0.46^*^ | 0.41^***^ | 0.47^***^ | 0.62^***^ | — |

*Latent factor correlations among the eight intelligence domains from the exploratory structural equation model*

*Note*. ^*^*p*<.05; ^**^ *p*<.01; ^***^ *p*<.001

**Table S4**

Monte Carlo evaluation of sample size for the eight-factor MISFOP ESEM and its associations with MoCA performance

|  |  |  |  | Power | | |  |
| --- | --- | --- | --- | --- | --- | --- | --- |
| *N* | Bias | Pattern recovered | Type I error | LI | LMI | INRI + SI | Ordinal check |
| 150 | 9.0% | 44.0% | 0.002 | 0.168 | 0.449 | 0.358 | 0.068 |
| 200 | 11.2% | 57.6% | 0.047 | 0.959 | 0.861 | 0.684 | 0.176 |
| 250 | 9.6% | 71.1% | 0.052 | 0.995 | 0.902 | 0.810 | 0.562 |
| 300 | 8.2% | 76.0% | 0.050 | 0.999 | 0.941 | 0.826 | 0.795 |
| 350 | 6.8% | 79.4% | 0.047 | 1.000 | 0.963 | 0.893 | 0.890 |
| 400 | 6.5% | 86.2% | 0.049 | 1.000 | 0.983 | 0.909 | 0.891 |
| 428 | 6.2% | 86.4% | 0.052 | 1.000 | 0.988 | 0.936 | 0.917 |
| 500 | 5.3% | 90.7% | 0.053 | 1.000 | 0.996 | 0.945 | 0.932 |
| 600 | 5.4% | 95.3% | 0.049 | 1.000 | 0.999 | 0.983 | 0.980 |

Note. Monte Carlo study following Muthén and Muthén (2002); 1,000 replications per sample size, all of which converged. The population model was the final MISFOP solution: the 29 × 8 ESEM pattern matrix including all cross-loadings (Table 4), the latent factor correlations (Table S3), and the standardized SET-ESEM associations with MoCA (Table 5). Each replication was analyzed with an eight-factor ESEM, and the resulting solution was rotated to best fit the population pattern (oblique target rotation) so that estimates could be matched to their population values; the substantive analysis used geomin rotation. LI = linguistic intelligence; LMI = logical-mathematical intelligence; INRI + SI = integrated intrapersonal and spiritual intelligence. Bias = largest relative bias across the 29 primary loadings. Pattern recovered = percentage of replications in which every item showed its highest loading on its intended factor. Type I error = mean empirical rejection rate across the eight structural paths in a separate set of replications in which every path was fixed to zero in the population (nominal *α* = .05). Power columns refer to the three associations that reached significance in the observed data (*β* = 0.36, 0.26 and 0.23). Ordinal check = lowest power across those three associations when simulated responses were categorized into five ordered categories matching the MISFOP response format. At *N* = 150 the rotated solution was unstable and the path estimates were markedly non-normal, which is reflected in both the depressed rejection rate and the erratic power at that sample size. Adequacy criteria (Muthén & Muthén 2002): bias < 10%, Type I error ≈ .05, power ≥ .80; the smallest sample examined that met all three was *N* = 250; the attained sample of the present study was *N* = 428. Sample sizes of 275, 325, 375 and 450 were also simulated and are omitted here for readability. Simulation code is available from the corresponding author.

**Table S5**

Measurement invariance of the 29-item, eight-factor MISFOP across sex and educational level

|  |  |  | Model fit | | | | Change in fit | | |
| --- | --- | --- | --- | --- | --- | --- | --- | --- | --- |
| Model | *χ²* | *df* | CFI | TLI | RMSEA | SRMR | ΔCFI | ΔRMSEA | ΔSRMR |
| *Sex* |  |  |  |  |  |  |  |  |  |
| Configural | 564.943 | 404 | 0.957 | 0.914 | 0.051 | 0.024 | — | — | — |
| Metric | 840.459 | 572 | 0.956 | 0.937 | 0.044 | 0.035 | -0.002 | -0.007 | +0.012 |
| Scalar | 887.461 | 593 | 0.948 | 0.929 | 0.046 | 0.037 | -0.008 | +0.003 | +0.002 |
| *Educational level* |  |  |  |  |  |  |  |  |  |
| Configural | 518.461 | 404 | 0.975 | 0.950 | 0.038 | 0.025 | — | — | — |
| Metric | 787.270 | 572 | 0.970 | 0.958 | 0.035 | 0.041 | -0.005 | -0.003 | +0.016 |
| Scalar | 855.037 | 593 | 0.959 | 0.944 | 0.041 | 0.043 | -0.011 | +0.005 | +0.002 |

Note. Multi-group exploratory structural equation models estimated with the MLR estimator and geomin rotation, matching the primary analysis; fit indices are the robust (MLR) values. Configural = no cross-group constraints; metric = factor loadings constrained to equality; scalar = loadings and item intercepts constrained to equality. Δ = change relative to the preceding model. Invariance criteria (Chen 2007): ΔCFI ≤ .010, ΔRMSEA ≤ .015, ΔSRMR ≤ .030. Women *n* = 343, men *n* = 85; educational level was dichotomized as secondary or below (*n* = 298) versus tertiary (*n* = 130). All models converged
