# Supplementary material for: Understanding Multiple Intelligences Among Older Adults: Development and Validation of the Multiple Intelligence Scale for Older People
Source: Brain Behav. 2026 Sep 23;16(9):e71762. doi: 10.1002/brb3.71762 (PMC13601738; doi:10.1002/brb3.71762)
Supplement: Supplementary file 2 [file BRB3-16-e71762-s002.docx]

**Appendix A**

*Modified back-translation model*

**Appendix B**

*The results of cultural equivalence on MISFOP.*

| **Items** | **The 35-item MIPQ IX** | **Items** | **The 36-item MISFOP** |
| --- | --- | --- | --- |
| 1. | Writing is a natural way for me to express myself. | 1. | Writing is my natural way of expressing myself. |
| 2. | At school, studies in English or social studies were easier for me than mathematics, physics and chemistry. | 2. | Knowledge in Mandarin or social studies was easier for me than mathematics, physics, and chemistry. |
| 3. | I have recently written something that I am especially proud of, or for which I have received recognition. | 3. | I have recently written something that I am particularly proud of or recognized for. |
| 4. | Metaphors and vivid verbal expressions help me learn efficiently. | 4. | Metaphors and vivid verbal expressions help me learn effectively. |
| 5. | At school, I was good at mathematics, physics or chemistry. | 5. | I am good at math, physics, and chemistry. |
| 6. | I can work with and solve complex problems. | 6. | I can solve complex problems, such as learning 3C products. |
| 7. | Mental arithmetic is easy for me. | 7. | I find mental arithmetic easy. |
| 8. | I am good at games and problem solving, which require logical thinking. | 8. | I am good at problem-solving and games that require logical thinking. For example: puzzle games, checkers, Gomoku, Chinese chess, bridge, mahjong, board games, and sudoku. |
| 9. | At school, geometry and other subjects involving spatial perception were easier for me than solving equations. | 9. | I am good at geometry and subjects that involve spatial perception. For example: Identify orientation on the street or the location on the floor. |
| 10. | It is easy for me to conceptualize complex and multidimensional patterns. | 10. | I can easily sort through complex and multifaceted problems. |
| 11. | I can easily imagine how a landscape looks from a bird’s-eye view. | 11. | I can easily imagine landscapes from a bird’s-eye view. |
| 12. | When I read, I form pictures or visual images in my mind. | 12. | When I read, I form pictures or visual images in my mind. |
| 13. | I am handy. | 13. | I can easily use my hands to complete practical tasks. For example: cooking, baking, gardening, assembling, repairing, knitting, or sewing. |
| 14. | I can easily do something concrete with my hands (e.g. knitting and woodwork). | 14. | I am good at demonstrating hands-on operations to others. |
| 15. | I am good at showing someone how to do something in practice. | 15. | I am good at physical activities or sports. For example: dancing, playing ball, running, or swimming. |
| 16. | I was good at handicrafts (e.g. woodwork; textiles) at school. | 16. | I am good at expressing ideas through body movements. |
| 17. | After hearing a tune once or twice I am able to sing or whistle it quite accurately. | 17. | After hearing a tune once or twice, I can sing or hum it accurately. |
| 18. | When listening to music, I am able to pick out individual instruments and recognize melodies. | 18. | I can distinguish the sound of each instrument and melody when I listen to music. |
| 19. | I can easily keep the rhythm when drumming a melody. | 19. | I can easily keep the rhythm when tapping out a melody. For example: having a good sense of rhythm. |
| 20. | I notice immediately if a melody is out of tune. | 20. | I can immediately detect when a melody goes off key. |
| 21. | Even in strange company, I can easily find someone to talk to. | 21. | I can easily find someone to talk to, even among a group of strangers. |
| 22. | I get along easily with different types of people. | 22. | I get along easily with different types of people. |
| 23. | I make contact easily with other people. | 23. | I can easily connect with others. |
| 24. | In negotiations and group work, I am able to support the group to find a consensus. | 24. | I help the group reach a consensus during teamwork and coordination. |
| 25. | I am able to analyze my own motives and ways of action. | 25. | I am able to analyze my own motivations and actions. |
| 26. | I often think about my own feelings and sentiments and seek reasons for them. | 26. | I often reflect on my feelings and emotions and look for their causes. |
| 27. | I regularly spend time reflecting on the important issues of life. | 27. | I often spend time reflecting on important issues in life. |
| 28. | I like to read psychological or philosophical literature to increase my self-knowledge. | 28. | I like to read psychological, philosophical, or religious literature to enhance self-knowledge. |
| 29. | In my busy everyday life I find it important to take time to think and reflect. | 29. | I find it important to spend time thinking and reflecting amid a busy daily life. |
| 30. | Even ordinary everyday life is full of amazing things. | 30. | Even ordinary, everyday life is full of interesting or novel things. |
| 31. | I often reflect on the meaning of life. | 31. | I often reflect on the meaning of life. |
| 32. | It is important to me to share a quiet moment with others. | 32. | I recognize the importance of sharing quiet moments with others. |
| 33. | I enjoy the beauty and experiences related to nature. | 33. | I like the beautiful scenery and experiences inherent in nature. |
| 34. | Protecting the environment is important to me. | 34. | I can quickly become familiar with the names and related information of natural features, plants, or animals. |
| 35. | I pay attention to what I consume in order to protect the environment. | 35. | To protect the environment, I am mindful of my consumption patterns. For example: using trash bags, reusable cups, or chopsticks. |
|  |  | 36. | I often pay attention to and collect information related to human development and historical sites. |
